# Isolation of Genes Involved in Biofilm Formation of a *Klebsiella pneumoniae* Strain Causing Pyogenic Liver Abscess

**DOI:** 10.1371/journal.pone.0023500

**Published:** 2011-08-12

**Authors:** Meng-Chuan Wu, Tzu-Lung Lin, Pei-Fang Hsieh, Hui-Ching Yang, Jin-Town Wang

**Affiliations:** 1 Department of Microbiology, National Taiwan University College of Medicine, Taipei, Taiwan; 2 Department of Internal Medicine, National Taiwan University Hospital, Taipei, Taiwan; National Institutes of Health, United States of America

## Abstract

**Background:**

Community-acquired pyogenic liver abscess (PLA) complicated with meningitis and endophthalmitis caused by *Klebsiella pneumoniae* is an emerging infectious disease. To investigate the mechanisms and effects of biofilm formation of *K. pneumoniae* causing PLA, microtiter plate assays were used to determine the levels of biofilm formed by *K. pneumoniae* clinical isolates and to screen for biofilm-altered mutants from a transposon mutant library of a *K. pneumoniae* PLA-associated strain.

**Methodology/Principal Findings:**

The biofilm formation of *K. pneumoniae* was examined by microtiter plate assay. Higher levels of biofilm formation were demonstrated by *K. pneumoniae* strains associated with PLA. A total of 23 biofilm-decreased mutants and 4 biofilm-increased mutants were identified. Among these mutants, a biofilm-decreased *treC* mutant displayed less mucoviscosity and produced less capsular polysaccharide (CPS), whereas a biofilm-increased *sugE* mutant displayed higher mucoviscosity and produced more CPS. The biofilm phenotypes of *treC* and *sugE* mutants also were confirmed by glass slide culture. Deletion of *treC*, which encodes trehalose-6-phosphate hydrolase, impaired bacterial trehalose utilization. Addition of glucose to the culture medium restored the capsule production and biofilm formation in the *treC* mutant. Transcriptional profile analysis suggested that the increase of CPS production in Δ*sugE* may reflect elevated *cps* gene expression (upregulated through *rmpA*) in combination with increased *treC* expression. In vivo competition assays demonstrated that the *treC* mutant strain was attenuated in competitiveness during intragastric infection in mice.

**Conclusions/Significance:**

Genes important for biofilm formation by *K. pneumoniae* PLA strain were identified using an in vitro assay. Among the identified genes, *treC* and *sugE* affect biofilm formation by modulating CPS production. The importance of *treC* in gastrointestinal tract colonization suggests that biofilm formation contributes to the establishment and persistence of *K. pneumoniae* infection.

## Introduction

*Klebsiella pneumoniae* is one of the most important pathogens causing opportunistic infections, such as pneumonia, sepsis, and inflammation of the urinary tract [1], [2]. In the past 20 years, the incidence of *K. pneumoniae*-caused community-acquired pyogenic liver abscess (PLA), complicated by meningitis and endophthalmitis, has increased globally [3]–[5]. Despite improved detection capacity and medical care, PLA is still a critical disease with high mortality rates [6]–[8]. Recent reports indicate that *K. pneumoniae* is the most frequent cause of PLA in Taiwan, Singapore, and Korea [7]–[11].

A bacterial biofilm is a complicated, community-like structure that comprises bacterial cells embedded in a self-produced exopolysaccharide (EPS) matrix. The biofilm is usually attached to inserted (e.g., stent) or living solid surfaces [12], [13]. Formation of a biofilm protects bacteria from attacks by phagocytosis and toxic molecules [13]–[15]. The inefficient penetration of antimicrobial oxidants and phagocyte-produced peptides into biofilms may result in the failure of immune systems to clear the bacteria [12]. In addition, the bacteria in biofilms are more tolerant of antibiotics than those in planktonic form. Indeed, the resulting resistance to antibiotics has been shown to hamper therapy [16]–[18].

Several factors required for biofilm formation have been identified in *K. pneumoniae* clinical isolates from the gastrointestinal tract and in strains that are associated with pneumonia and urinary tract infection [19]–[22]. A study using signature-tagged mutagenesis and surfaces coated with human extracellular matrix (HECM) identified a protein involved in capsule biosynthesis that is essential for biofilm formation by *K. pneumoniae* [21]. A recent study showed that capsule genes *wza* and *ORF14* are important to early stage biofilm formation by *K. pneumoniae* [22]. However, the regulatory mechanism of biofilm formation in *K. pneumoniae* PLA strains remains unclear. Therefore, we compared biofilm formation between community-acquired PLA-associated and non-tissue-invasive *K. pneumoniae* strains. This work included screening for biofilm-related genes using a mutant library constructed in a clinical *K. pneumoniae* PLA strain, and further characterizing the roles in biofilm formation of the identified genes.

## Materials and Methods

### Ethical treatment of animals

BALB/cByl mice were bred and housed in specific pathogen–free rooms within the animal care facilities of the Laboratory Animal Center at the National Taiwan University College of Medicine (NTUCM) with free access to food and water. All procedures were approved by the NTUCM and College of Public Health Institutional Animal Care and Use Committee (IACUC approval number: 20060139), and followed the recommendations of the *Guide for the Care and Use of Laboratory Animals* of the National Institutes of Health and the Taiwanese Animal Protection Act.

### Bacterial strains, plasmids, and culture conditions

The bacterial strains and plasmids used in this study are listed in Table 1. A total of 74 clinical isolates of *K. pneumoniae* were cultured from blood samples collected at National Taiwan University Hospital (NTUH) between 1997 to 2003, as described previously [4], [23]. Of these strains, 42 were isolated from patients with PLA (PLA-associated); the remaining 32 were isolated from patients with sepsis but without PLA or other metastatic infections in other tissue (non-tissue-invasive). *K. pneumoniae* and *Escherichia coli* strains were grown in Luria-Bertani (LB) medium, supplemented (as needed) with 50 µg/mL kanamycin or 100 µg/mL chloramphenicol.

**Table 1:** Bacterial strains and plasmids used in this study.

Strains or plasmids	Description	Source
**Bacteria**		
*K. pneumoniae* strains		
*K. pneumoniae* isolates (74)	Clinical isolates collected from National Taiwan University Hospital during 1997–2003	[4], [23]
NTUH-K2044	Clinically isolated strain causing PLA, the parental strain for generation of isogenic mutants	[30]
Δ*sugE*	NTUH-K2044 isogenic mutant with deletion of *sugE* gene	This study
Δ*sugE*/*sugE*+	NTUH-K2044 Δ*sugE* with *sugE* cassette between *pgpA* and *yajO*	This study
Δ*treC*	NTUH-K2044 isogenic mutant with deletion of *treC* gene	This study
Δ*treC*/*treC*+	NTUH-K2044 Δ*treC* with *treC* cassette between *pgpA* and *yajO*	This study
Δ*wza*	NTUH-K2044 isogenic mutant with deletion of *wza* gene	This study
Δ*treB*	NTUH-K2044 isogenic mutant with deletion of *treB* gene	This study
Δ*lacZ*p	NTUH-K2044 isogenic mutant with deletion of *lacZ* promoter	[28]
*E. coli* strains		
DH10B	F^−^ *mcrA* Δ(*mrr*-*hsdRMS*-*mcrBC*) Φ80 δλαχZ ΔM15 Δ*lacX*74 *endA*1 *recA*1 *deoR* (*ara leu*)7697 *ara*Δ139 *galU galK nupG rpsL* λ^−^	Invitrogen
**Plasmids**		
pGEM-T easy	TA cloning vector	Promega
pKO3-Km	pKO3-derived plasmid, with a kanamycin-resistant cassette inserted in *AccI* site	[26]
pKO3-Km-*pgpA*-*yajO*	pKO3-Km derived plasmid, with a region containing part of the *thiL*, the *yajO*, and part of the phosphatidyl- glycerophosphatase A genes inserted into *Not*I site	[28]
pPCR2.1-TOPOII-GFP-CAT	GFP-expressing plasmid	[30]

NOTE. *K. pneumoniae*, *Klebsiella pneumoniae*; *E. coli*, *Escherichia coli*; PLA, pyogenic liver abscess; Km, kanamycin; GFP, green fluorescence protein.

### Biofilm assay

The microtiter plate assay developed by O'Toole and Kolter was modified to examine biofilm formation by mutants [24]. Briefly, 1 µL of an overnight culture was inoculated into 100 µL of fresh LB broth in each well of a 96-well polystyrene plate. After static incubation at 37°C for 5 hours, bacteria were stained with 25 µL of 0.5% crystal violet for 20 min. The plate then was washed with deionized water, the biofilm-bound dye was eluted with 95% ethanol, and the absorbance was measured at 550 nm.

### Inverse polymerase chain reaction (PCR) and DNA sequencing

To analyze the transposon-insertion site of *K. pneumoniae* mutants, the genomic DNA of the bacteria was extracted using phenol-chloroform method, completely digested with *Pst*I (New England Biolabs; NEB), and then self-ligated with T4 DNA ligase (NEB). The fragments containing both ends of the transposon and the flanking region of the insertion site were amplified by inverse PCR using primers Km4180F and Km2921R (Table S1) and then subjected to DNA sequencing.

### Mucoviscosity assay

The mucoviscosity levels were determined by centrifugation [25]. *K. pneumoniae* NTUH-K2044 and its transposon mutants were cultivated at 37°C overnight. Aliquots of 1 mL of bacteria were pelleted at 12,000× g for 10 min.

### Capsular polysaccharide (CPS) extraction and measurement

zCPS of *K. pneumoniae* was purified using the hot phenol-water method [4]. A total of 1×10^9^ colony forming units (CFU) of bacteria were harvested and suspended in 150 µL deionized water. An equal volume of saturated phenol was added and the mixture was incubated at 65°C for 20 min. The mixture was extracted with 150 µL chloroform and vortexed intensely. After centrifugation, the supernatants were collected. The amount of surface polysaccharide was determined by the phenol-sulfuric acid assay [15]; CPS was quantified by monitoring the absorbance at 490 nm using fucose as a standard.

### Construction of deletion mutants of *K. pneumonia*

The flanking regions of the target genes *treC*, *treB*, *sugE*, and *wza* were amplified by PCR with gene-specific primers (Table S1), and the resulting fragments were cloned into the temperature-sensitive vector pKO3-Km, which contains a kanamycin resistance gene and a *sacB* gene for positive and negative selection, respectively [26], [27]. The resulting plasmid was transformed into strain NTUH-K2044 and plated at the non-permissive temperature (43°C) to force integration of the plasmid into the bacterial chromosome by single crossover. During subsequent culturing, cells were grown at the permissive temperature (30°C) in the presence of sucrose and absence of kanamycin, selecting for plasmid excision and loss. The resulting colonies were screened by PCR for deletion of the target gene [27].

### Construction of chromosomal complementation strains of *K. pneumonia*

To generate the chromosomal complementation strains, the *treC* or *sugE* gene cassette was amplified with gene-specific primers (Table S1) and cloned into the plasmid pKO3-Km-*pgpA*-*yajO* [28]. The resulting plasmid was transformed into the corresponding mutant and passaged as above to generate gene replacements. PCR screening was used to confirm the insertion of the *treC* or *sugE* gene cassette into the intergenic region between the *pgpA* and *yajO* open reading frames, as previously described [28].

### Slide culture

For each well of a 24-well plate, the bottom of the well was layered with glass beads, and a piece of slide glass was placed on the beads; a volume of medium just sufficient to submerge the slide was added. A plasmid encoding green fluorescent protein (GFP) and carrying a chloramphenicol resistance cassette was introduced into each *K. pneumoniae* strain by electroporation. The production of GFP was confirmed under excitation by ultraviolet light. *gfp*-expressing bacteria were cultivated overnight and inoculated into each well in fresh LB broth at a ratio of 1∶100. The slides were harvested at the indicated time points and washed twice with 1× phosphate-buffered saline (PBS). The confocal fluorescence images of biofilms were observed using an argon ion laser (488 nm; Leica TCS-SP5). The images were processed with Bitplane Imaris to generate the xy-, xz-, and yz- dimensional images.

### RNA isolation and real-time RT-PCR

Log-phase *K. pneumoniae* NTUH-K2044 wild-type strain and the mutants were harvested, and total RNA was extracted from 1 mL of culture using the RNeasy Mini Kit (Qiagen) according to the manufacturer's instructions. Aliquots of 400 ng of total RNA were converted into complementary DNA (cDNA) using SuperScript® II Reverse Transcriptase (Invitrogen). Gene expression levels were monitored by real-time RT-PCR using Platinum SYBR Green qPCR SuperMix-UDG in an ABI7900 thermocycler (Applied Biosystems). The cycle threshold (Ct) of 23S rRNA from the same sample was used to normalize the ΔCt (calculated threshold cycle) of the target gene. The relative quantities of RNA were obtained by calculation of the fold-change with the 2^−ΔΔCt^ method [29].

To analyze the gene expression differences between biofilm and planktonic cells, NTUH-K2044 was grown on glass slides or cultivated with shaking at 37°C. The glass slides were washed twice with 1×PBS to remove unattached cells, and bacteria then were collected at 4, 8, 16, 24, 48, or 72 hours by vigorous shaking followed by centrifugation. For planktonic cells, 1-mL aliquots of bacteria from shaking cultures were harvested by centrifugation. The relative amounts of RNA were calculated by comparing the expression in biofilm cells to that in planktonic cells collected at the same time point.

### In vivo competition assay

To determine the competitiveness of the *K. pneumoniae* NTUH-K2044 isogenic mutant, we used a deletion mutant of the *lacZ* promoter (Δp*lacZ*) that had been constructed and described in a previous study [28]. When grown on agar plates containing 1 mmol/L isopropyl β-D-1-thiogalactopyranoside (IPTG) and 50 mg/mL X-gal, Δp*lacZ* exhibits a white colony phenotype, while the Δ*treC* mutant exhibits a blue colony phenotype. The wild-type or the Δ*treC* strain and the Δp*lacZ* strain were combined 1∶1 (1×10^6^ CFU each) in 0.1 mL of 0.95% saline solution, and the mixture was inoculated intragastrically into 5-week-old female BALB/cByl mice. Mice were sacrificed on the seventh day after inoculation. The colons were harvested and homogenized in 1×PBS; serial dilutions (in 1×PBS) were plated to solid medium to recover the colonizing bacteria. *K. pneumoniae* strains (which were easily differentiated from other aerobic flora by colony morphology) were randomly picked for further screening, including confirmation of species identity by PCR with primers from the 23S rRNA gene of *K. pneumoniae*. The competitive index (CI), defined as the input and output ratio of the test strain to the Δp*lacZ* strain, was determined by plating bacteria onto LB indicator plates containing IPTG and X-gal. The CI was regarded as the ability of the respective strain to colonize mice.

### Statistical Analyses

Data are presented as means ± standard deviations (SDs). Statistical significance of comparisons of mean values was assessed by a two-tailed Student's *t* test using Prism 5 (Graphpad) software. In the case of CI, mean values were assessed by Wilcoxon signed rank test. P values of <0.05 were considered significant.

## Results

### Biofilm formation by *K. pneumoniae* clinical isolates

**Figure 1:**
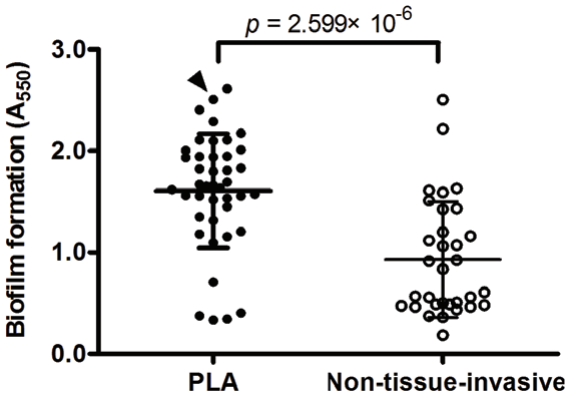
Biofilm formation of *K. pneumoniae* clinical isolates. Overnight cultures of *K. pneumoniae* PLA and non-tissue-invasive strains were grown in fresh Luria-Bertani broth at a ratio of 1∶100 in polystyrene plates at 37°C for 5 hr. The sessile bacteria were stained with crystal violet, washed to remove unbound cells, eluted with 95% ethanol, and monitored the absorbance at 550 nm. Data shown are the means of 3 independent experiments (duplicate in each experiment), and error bars shows the standard deviation (SDs). *p* = 2.599×10^−6^, by Student's *t*-test. Arrow head, the absorbance values (A_550_) of NTUH-K2044.

Using a polystyrene microtiter plate assay, we examined seventy-four clinical isolates of *K. pneumoniae*, including 42 PLA-associated and 32 non-tissue-invasive strains, for the ability to form biofilms. The biofilm-forming abilities of the PLA-associated isolates were significantly higher than those of the non-tissue-invasive isolates: the values for crystal violet staining were 1.606±0.562 vs. 0.928±0.572, respectively (*p* = 2.599×10^−6^, by Student's t-test) (Fig. 1).

### Biofilm-related genes in *K. pneumoniae* NTUH-K2044

**Figure 2:**
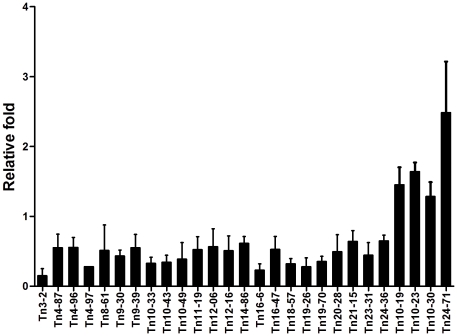
Biofilm formation of transposon mutants of *K. pneumoniae* NTUH-K2044. Overnight cultures of *K. pneumoniae* PLA and non-tissue-invasive strains were grown in fresh Luria-Bertani broth at a ratio of 1∶100 in polystyrene plates at 37°C for 5 hr. The sessile bacteria were stained with crystal violet, washed to remove unbound cells, eluted with 95% ethanol, and monitored the absorbance at 550 nm. Data are representative of three independent experiments (duplicate in each experiment).

To identify genes associated with biofilm formation, we chose to focus on the clinical PLA strain NTUH-K2044, which displayed strong biofilm growth (indicated by arrow head in Fig. 1; A_550_ = 2.507±0.421) in the polystyrene microtiter plate assay and was highly amenable to genetic modification. In previous work [30], we described the construction of a library of mini-Tn5 transposon-insertion mutants in *K. pneumoniae* NTUH-K2044. In the present study, we assessed biofilm formation in a total of 2,500 of these insertion mutants, with the goal of identifying genes associated with biofilm formation. Mutants with two kinds of phenotypes were obtained: twenty-three mutants showed decreased biofilm formation, and four mutants showed enhanced biofilm formation (Fig. 2). The growth rates of all of these mutants were similar to that of the wild-type strain.

We determined the transposon insertion sites by inverse PCR and DNA sequencing; the resulting sequences were analyzed by comparison (using the BLASTX program) to the full genome sequence of NTUH-K2044 (accession number AP006725). The results are shown in Table S2. Among the 23 mutants exhibiting a reduced-biofilm phenotype, four categories of insertion targets could be discerned. Seven mutants were affected in genes involved in cellular processing and signaling (a gene encoding a putative secretion ATPase; *pmrD*; *fdhF*; *clpX*; a gene encoding a LuxR family regulator; an exonuclease encoding gene; and *cas3*, invovled in the CRISPR bacterial immunity system). Four transposon mutants had insertions in genes associated with surface molecules, including capsule, adhesion polysaccharide, and pilin (*wza*, *pgaA*, *wzc*, and *fim* genes, respectively). Six mutants were disrupted in genes encoding hypothetical proteins of unknown function. The remaining six mutants were disrupted in genes involved in carbohydrate transport or metabolism (the gene encoding 6-phospho-beta-glucosidase A; *celB*; *dgoT*; and 3 *treC* mutants). The three *treC* mutants were disrupted at the same site. Among the mutants with biofilm-increased phenotype, sequence analysis for mutant Tn10-23 indicated the insertion of a transposon in the ribosomal binding site of a small multidrug resistance gene, *sugE*. In the mutant Tn24-71, the transposon was located 706 bp upstream of *cspC*, which encodes a cold-shock protein gene. Mutants Tn10-19 and Tn10-30 were disrupted in proteins of unknown-function.

### Effects on the production of CPS

**Figure 3:**
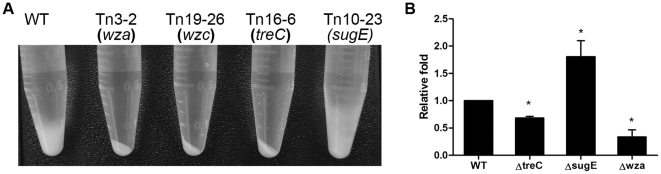
Mucoviscosity, capsular polysaccharide (CPS) production and biofilm formation of *K. pneumoniae* NTUH-K2044 and isogenic mutants. A, *K. pneumoniae* NTUH-K2044 and the transposon mutants Tn3-2 (*wza*), Tn19-27 (*wzc*), Tn16-6 (*treC*), and Tn10-23 (*sugE*) grown overnight were pelleted at 12,000 g for 10 min. B, The amount of CPS production nof the wild-type strain NTUH-K2044, *treC*, *sugE*, and *wza* mutants were determined by the sulfuric acid assay. *, *p*<.05. Data are representative of three independent experiments (triplicate in each experiment).

Because the capsule has been known to play a role in biofilm formation, we determined the mucoviscosity phenotype of the *K. pneumoniae* NTUH-K2044 wild-type strain and the transposon mutants by centrifugation [25]. Due to the thick and mucoid capsule, it is difficult to pellet encapsulated *K. pneumoniae* cells by centrifugation. In contrast, capsule mutants have a reduced mucoviscosity phenotype, facilitating pelleting of cultures of such bacterial mutants [25]. As expected, mutants of known capsule genes *wza* (mutant Tn3-2) and *wzc* (mutant Tn19-26) exhibited reduced mucoviscosity. We additionally observed that the *treC* mutant (Tn16-6) showed reduced mucoviscosity (Fig. 3A). By comparison, it was more difficult to pellet the *sugE* transposon mutant (Tn10-23) than to pellet the wild-type strain. The mucoviscosity of the other 21 transposon mutants was similar to that of the wild-type strain. We further quantified the amount of CPS production of the mutants by the sulfuric acid assay. This test showed that the *treC* mutant synthesized less CPS than the wild-type strain, whereas the *sugE* mutant produced more CPS (Fig. 3B). Thus, the remainder of the study focused on the role of *treC* and *sugE* on biofilm formation and capsule production.

### Construction and characterization of treC and sugE deletion and complementation strains

**Figure 4:**
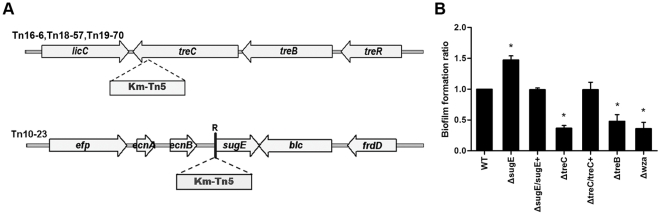
Schemas of transposon-insertion site and biofilm formation of *K. pneumoniae* NTUH-K2044 and isogenic mutants. A, Schemas of transposon-insertion site of *treC* mutant (Tn16-6, Tn18-57, and Tn19-70) and *sugE* mutant (Tn10-23). Broad arrows, the locations and orientations of the open-reading frames. R, the ribosomal binding site. B, Biofilm formation of *K. pneumoniae* NUTH-K2044, the deletion mutants, and the complementation strains. Bacteria were grown at 37°C in polystyrene plates containing fresh Luria-Bertani broth for 5 h. The sessile bacteria were stained with crystal violet, washed to remove unbound cells, eluted with 95% ethanol, and monitored the absorbance at 550 nm. *, *p*<.05. Data are representative of three independent experiments (triplicate in each experiment).

Since *treC* is the downstream gene of the *treBC* operon and the *sugE* gene is a single-gene operon, tranposon insertions in these loci were considered unlikely to be exerting polar effects in these mutants (Fig. 4A). Nevertheless, we constructed deletion and complementation strains for these two genes to confirm our results and to rule out the possibility of polar effects or the presence of spontaneous mutations at other loci. Indeed, deletion of *treC* resulted in a significant reduction in biofilm production (Fig. 4B, Δ*treC*), whereas deletion of *sugE* enhanced the ability to produce biofilm approximately 1.5-fold (Fig. 4B, Δ*sugE*), consistent with the biofilm phenotypes of the respective transposon mutants. Chromosomal complementation restored the ability to produce as much biofilm as the wild-type strain (Fig. 4B, Δ*treC*/*treC*+ and Δ*sugE*/*sugE*+), confirming that the defects were in fact locus specific.

In *E. coli*, *treC* and *treB*, which encodes trehalose-specific phosphotransferase system enzyme IIB/IIC component (the EII^Tre^ protein), form an operon that is controlled by the TreR regulatory protein [31]. We generated a *K. pneumoniae treB* deletion mutant and observed that Δ*treB* also exhibited reduced biofilm formation (Fig. 4B, Δ*treB*). Together, these results suggested that the function of the *treBC* operon is important for biofilm formation in *K. pneumoniae*.

### Biofilm formation on glass surface by treC and sugE mutants

**Figure 5:**
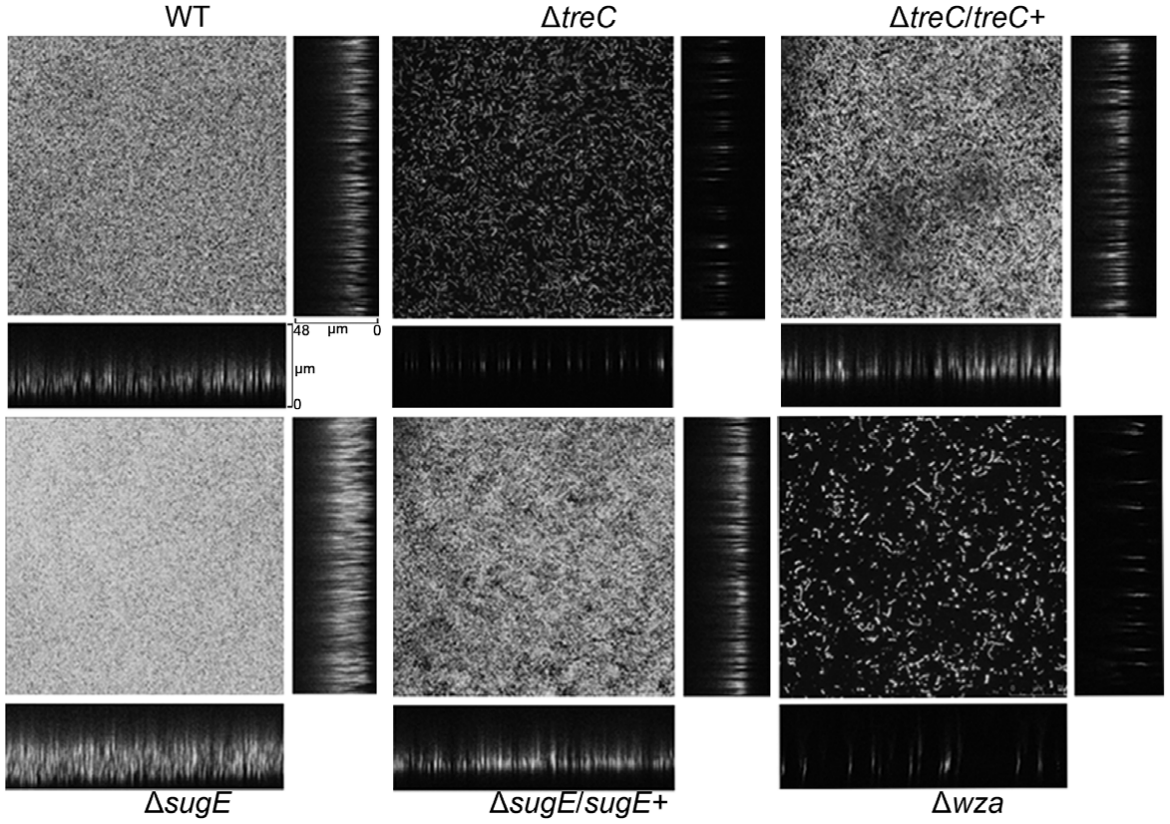
Biofilm formation by *K. pneumoniae* NTUH-K2044 and its isogenic mutants. Cover slides were placed into 24-well plates, which had been paved with glass beads. GFP-expressing *K. pneumoniae* strains were cultured overnight, inoculated into fresh LB for 18 h. The cover slides were washed with 1×PBS twice while shaking. The fluorescent signals were examined with a confocal microscope under 400×. The photographs depicted mimic images through the *x–y* plot (central), the y–z plot (right), and the x–z plot (below) of the biofilms. Data represent one of three independent experiments.

Using confocal microscopy, we characterized biofilm formation on glass slides by the parent and the deletion mutants. The biofilm of the wild-type strain was observed at 18 hr after inoculation (Fig. 5, WT). In this biofilm, the cell-to-cell spaces were presumably filled with matrix. The bacteria were stacked compactly, forming a 30-µm-thick multi-layered structure; it was difficult to distinguish the outline of a single cell within the complex. In the Δ*treC* culture, bacterial cells were scattered in a disorderly manner over the glass surface; no further three-dimensional structure could be observed (Fig. 5, Δ*treC*). In the Δ*sugE* mutant, the bacteria formed an extremely compact and thick structure (Fig. 5, Δ*sugE*). There was little empty space between the cells. The depth of the biofilm formed by Δ*sugE* extended approximately 42 µm. Complementation of the *treC* and *sugE* mutations by the respective genes restored biofilm formation on glass slides (Fig. 5, Δ*treC*/*treC*+ and Δ*sugE*/*sugE*+). In addition to the *treC* and *sugE* mutants, we also randomly selected other transposon mutants for examination of biofilm formation on glass slides. For all of the tested strains, biofilm formation also was characterized by confocal microscopy following growth on polystyrene microtiter plates (data not shown). The results with polystyrene surfaces were consistent with those observed on glass slides for the respective strains.

### Biofilm development by ΔtreC

**Figure 6:**
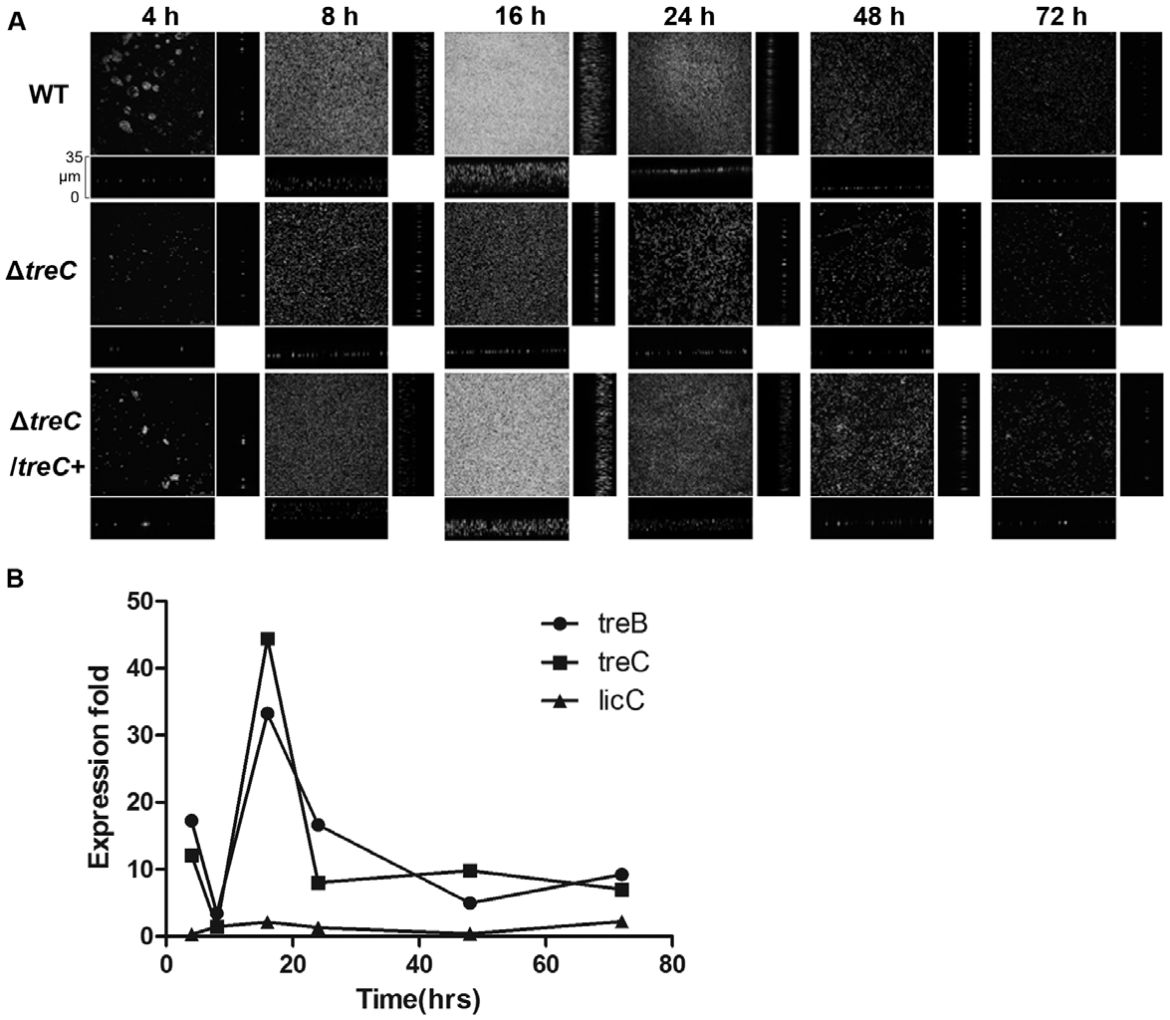
Biofilm development of *K. pneumoniae* Δ*treC* on glass slides and the comparison of the *treBC* expression differences between biofilm and planktonic cells. A, *K. pneumoniae* NTUH-K2044 (WT), Δ*treC*, Δ*treC*/Δ*treC*+ were grown at 37°C on glass slides, harvested at 4, 8, 16, 24, 48, and 72 h, and observed with a confocal microscope under 400× oil lens. The 177 sections obtained at 0.2 µm intervals were processed using Bitplane Imaris. B, The wild-type strains which were grown under planktonic culture condition or under biofilm slide culture condition were harvested at 4, 8, 16, 24, 48, and 72 h. The gene expression was determined by real-time RT-PCR. The data showed that the fold change in the expression of *treB* and *treC* gene in the biofilm cells with respect to that of in the planktonic cells. The expression of *licC* was taken as an internal control. Data represent one of two independent experiments.

To investigate which step of biofilm formation was influenced by deletion of *treC*, we observed the biofilm structure formed on glass slides during the first 72 hours of growth. The wild-type strain formed microcolonies at the initial stages. The thickness of the biofilm then increased, with the structure maturing from 4–16 h, before dissociating starting at 24 h (Fig. 6A). Although the Δ*treC* strain could attach onto the surface at 4 h, the mutant did not form microcolonies. The attached bacteria increased in number by 8 h, but no advanced architecture was observed at the following stages. The attached cells also disassociated from the surface after 16 h. To further characterize the role of *treC* in biofilm formation, we grew the wild-type strain either as biofilms on glass slides or as planktonic cultures; collected the cells at 4, 8, 16, 24, 48, and 72 hours; and compared (using real-time RT-PCR) the expression of *treC* and *treB* in the two types of culture conditions. In biofilm cells, the expression of *treC* and *treB* rose from 8 h, increasing (at 16 h) to 44-fold (*treC*) and 33-fold (*treB*) the levels seen in planktonic cells. Expression of these genes then descended at subsequent time points (Fig. 6B). The expression of *licC*, the gene adjacent to *treC*, served as an internal control. This gene, which has not been implicated in biofilm formation, did not exhibit significant changes during biofilm formation or during planktonic growth. These observations suggest an important role for *treC* during biofilm development.

### Role of treC in CPS production

**Figure 7:**
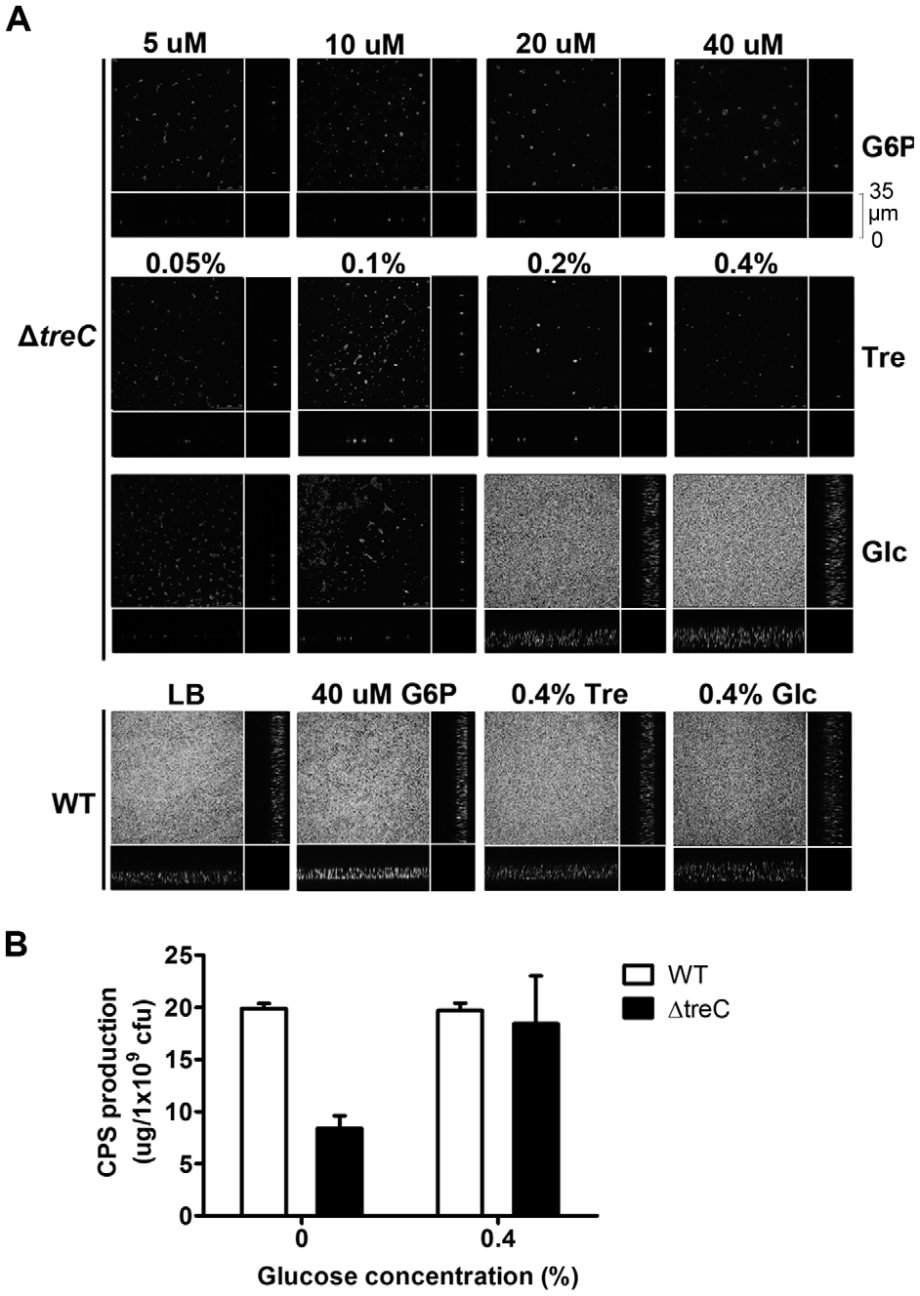
Biofilm and capsular polysaccharide production of Δ*treC* in Luria-Bertani broth supplemented with glucose. A, Biofilm formation by *K. pneumoniae* NTUH-K2044 and Δ*treC* on glass surface. GFP-expressing *K. pneumoniae* strain was grown on glass cover slide and harvest at 16 h. The cover slides were washed with PBS twice while shaking. The fluorescent signals were observed with a confocal microscope using oil immersion under 400×. The 177 sections obtained at 0.2 µm intervals were processed using Bitplane Imaris. Data represent one of two independent experiments. B, The capsular polysaccharide of wild-type strain (WT) and Δ*treC* grown in Luria-Bertani (LB) broth, or LB broth supplemented with 0.4% of glucose were extracted and determined by sulfuric acid assay. *, *p*<0.05. Data shown are mean from three independent experiments.

The deletion of *treC* led to decreased capsule production. Therefore, we also examined the transcriptional profile of the Δ*treC* strain by DNA microarray analysis. Notably, the expression levels of *cps* genes in Δ*treC* did not differ significantly from those in the wild-type strain. In addition, few genes were affected at the transcriptional level in Δ*treC* (data not shown). We therefore examined the function of the *treC* gene product. In *E. coli*, *treC* encodes trehalose-6-phosphate hydrolase, which catalyzes the conversion of trehalose-6-phosphate to glucose and glucose-6-phosphate (G-6-P) [32]. The *K. pneumoniae* Δ*treC* mutant could not grow on minimal (MMA) plates supplemented with trehalose as the sole carbon source (data not shown), indicating that the function of the TreC protein in *K. pneumoniae* is identical to that in *E. coli*. Both of the screening experiments and the follow-up studies described above were performed in LB medium. Therefore, we next cultivated Δ*treC* in LB supplemented with glucose, trehalose, or G-6-P and observed the cultures by confocal microscopy. Biofilm formation by Δ*treC* was observed only in medium supplemented with glucose, but not in medium supplemented with trehalose or G-6-P (Fig. 7A). In contrast, there was no significant difference in biofilm formation by the wild-type strain when cultivated in LB medium or in LB medium supplemented with trehalose, G-6-P, or glucose. We also measured the production of CPS under these different culture conditions. Consistent with the results of the biofilm assay, the level of CPS in the Δ*treC* mutant was elevated in the presence of glucose, while that of the wild-type strain did not show a similar increase (Fig. 7B).

### Upregulation of the cps gene cluster in ΔsugE

**Figure 8:**
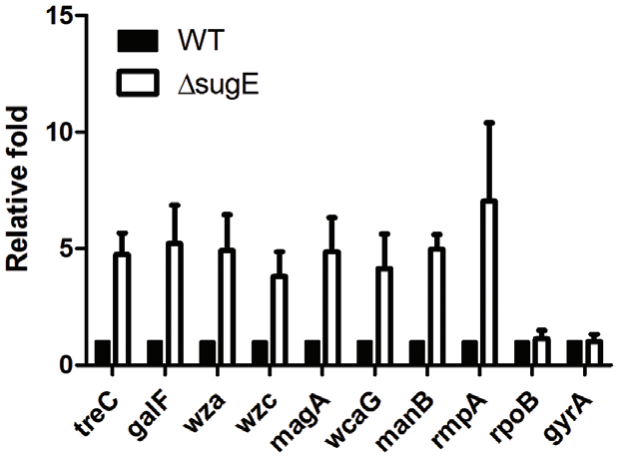
The transcriptional levels of *treC* and capsule-associated genes in the *sugE* mutant. Total RNA was extracted from *K. pneumoniae* NTUH-K2044 wild-type strain (WT) and Δ*sugE* cultivated in LB broth. The expression level of each gene in wild-type strain was taken as basal levels to obtain the value of relative level in Δ*sugE* strain. Data shown are the means of 3 independent experiments, and error bars shows the SDs.

To understand the mechanism of modulation of biofilm formation by *sugE*, a DNA microarray [26] was used to compare the transcriptional profile of Δ*sugE* with that of NTUH-K2044 (data not shown). Surprisingly, *treC* gene showed the greatest fold increase of mRNA expression in the Δ*sugE* mutant. In addition, *cps* genes also had higher expression levels in Δ*sugE*. We confirmed the RNA expression levels of these genes by quantitative PCR. Besides the *treC* gene, CPS-associated genes *galF*, *wza*, *wzc*, *magA*, *wcaG*, and *manB* displayed higher expression levels in the Δ*sugE* mutant compared to those of the wild-type strain (Fig. 8). Moreover, *rmpA*, a gene encoding a known regulator of the capsular gene cluster, also was induced in Δ*sugE*. The expression of two housekeeping genes, *rpoB* and *gyrA*, did not differ significantly between the wild-type strain and Δ*sugE*, providing an internal control. These results suggested that the increase of CPS production in Δ*sugE* may reflect elevated *cps* gene expression (upregulated through *rmpA*) in combination with increased *treC* expression.

### Animal study

**Figure 9:**
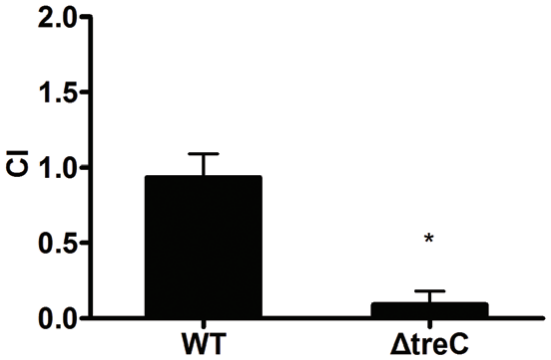
The in vivo competitiveness of Δ*treC*. Groups of 6 BALB/c mice were fed with 2×10^6^ *K. pneumoniae*. (Δp*lacZ* strain mixed with the wild-type strain or the Δ*treC* strain, the ratio was 1∶ 1). The bacteria were harvested from colon when mice were euthanized on the 7^th^ day after infection and plated onto LB plates. The ratio of *lacZ*-negative colonies to *lacZ*-positive colonized was determined. Competition index of Δ*treC* group vs Δp*lacZ* mutant group, 0.083±0.25, *p = 0.0039.

[28]. We determined the in *vivo* competitiveness of the Δ*treC* strain by comparing the *in vivo* intestinal colonization ability of Δ*treC* and Δp*lacZ*, which (as described previously) provides a colony-color assay for the *in vivo* competition assay [28]. The competitiveness of the Δp*lacZ* strain was similar to that of the wild-type strain (CI = 0.93), whereas the CI of the Δ*treC* strain decreased to a value of approximately 0.1 (Fig. 9). These results suggested that the deletion of *treC* impairs the ability of *K. pneumoniae* to compete *in vivo*.

## Discussion

To survive and cause a subsequent infection in the host, pathogenic enterobacteria need to colonize their hosts. They achieve this by developing biofilms while defending themselves against host immunity. In this study, biofilm formation was shown to be increased in PLA strains compared to non-tissue-invasive strains. The results suggest that biofilm formation may play a role in PLA pathogenesis, although the mechanism awaits further investigation. Additionally, we screened a transposon mutant library of a *K. pneumoniae* PLA strain NTUH-K2044; using a microtiter plate assay, we identified genes related to biofilm formation. Subsequently, static cultures on glass slides were used to verify the results from the microtiter plate assay, demonstrating that the alterations in adherence to the polystyrene surface by the transposon mutants were correlated with the formation of biofilms on the glass surface. The consistent results obtained via these two assays confirm that these genes play an important role in biofilm formation.

Several previous works have identified biofilm-related genes in *K. pneumoniae* strains that caused pneumonia, urinary tract infections, and nosocomially acquired infections of the gastrointestinal tract [19]–[22], [33]. However, none of these studies examined biofilm formation by *K. pneumoniae* PLA strains. In the present study, we identified genes involved in the biosynthesis of surface molecules required for the formation of biofilms, such as capsule, poly-beta-1,6-N-acetyl-D-glucosamine (PGA), and pilin. The importance of capsule, PGA, and pilin on biofilm formation was in agreement with previous reports [21], [22], [34], [35]. Contributions of sugar-specific phosphotransferase systems, ClpX, and LuxR-family regulators were also observed in *K. pneumoniae* and other organisms [21], [36], [37]. The present work also implicated other genes, such as *cspD*, *pmrD*, *fdhF*, and loci encoding CRISPR-associated proteins, in biofilm formation for the first time. The proposed functions of these genes were based solely on sequence analysis here; the detailed functions and contributions of these genes in biofilm formation will require further experiments.

Previous studies demonstrated the correlation of CPS production with biofilm formation by *K. pneumoniae*. Boddicker and colleagues showed that K2 capsule *ORF8* was essential for biofilm formation on a surface coated with human extracellular matrix material [21]. Mutations in *wza* and *ORF14* resulted in decreased adherence at the initial stage of biofilm formation by *K. pneumoniae* strain LM21 [22]. In our study, mutants with transposon insertions within the CPS loci *wza* and *wzc* were deficienct in biofilm formation, a result that is in agreement with the previous observations [22]. We also identified two genes (*treC* and *sugE*) outside the *cps* region. Mutations in these genes not only affected biofilm formation but also influenced bacterial mucoviscosity and CPS production.

Among the transposon-insertion mutants showing reduced biofilm formation, three mutants were disrupted in *treC*. Deletion of *treC* not only impaired trehalose utilization but also resulted in a reduction in capsule production. Deletion of *treB* (which is predicted to share function and regulation with *treC*) resulted in the same reduced-biofilm phenotype. Confocal microscopy was employed to demonstrate that the *treC* mutant could not form advanced biofilm structures starting from 4 h of development. Separate experiments showed that wild-type cells that were forming biofilms were elevated for expression of *treB* and *treC*. The expression of *treBC* increased during biofilm development before subsequently declining when biofilm cells entered the dispersal stage. Notably, induction of *treBC* was not seen in wild-type cells growing in the planktonic phase. These results imply that transportation and catalysis of trehalose contributes to biofilm establishment, and apparently not to the dispersal of mature biofilm.

By analogy to the homologous proteins in *E. coli*, the EII^Tre^ protein (encoded by *treB*) mediates the uptake of trehalose and phosphorylates the sugar to form trehalose-6-phosphate. The trehalose-6-phosphate hydrolase (encoded by *treC*) splits trehalose-6-phosphate into glucose and glucose-6-phosphate. These two molecules then enter into the glycolytic pathway. The deficiency of *K. pneumoniae* Δ*treC* may influence the production of energy and so impair bacterial growth during biofilm establishment. If so, medium supplementation with glucose and G-6-P would be expected to restore biofilm production in the Δ*treC* mutant. However, our data showed that the addition of glucose, but not that of G-6-P, rescued the deficiency of biofilm formation of Δ*treC*, indicating that the energy deficiency was not the main cause of the reduction of biofilm. Instead, we propose that *treC* is vital for biosynthesis of the matrix required for biofilm formation. The CPS from *K. pneumoniae* serotype K1 is composed of glucose, fucose, and glucouronic acid [38]. Our results showed that supplementation with glucose restored both biofilm formation and CPS production in the *treC* mutant. These results suggest that *treC* might promote the production of CPS by providing a structural component (glucose) required for biofilm formation.

Results of the deletion and complementation of *sugE* supported the observed phenotype of the transposon mutant. In addition, we observed increased expression of the *cps* region, *rmpA*, and *treC* in the *sugE* mutant. RmpA is a regulator of CPS biosynthesis, activating capsule production in an RcsB-dependent manner [39]. Thus, the increased production of CPS in Δ*sugE* may be induced via the transcriptional activity of RmpA and the increased expression of *treC*. In *E coli*, the *sugE* homolog is predicted to encode an inner-membrane protein with a very short tail facing the cytoplasm [40]; such a protein is unlikely to serve as a direct regulator of transcription. Also in *E. coli*, osmotic shock has been shown to increase capsule synthesis via transcriptional regulation [41]. We propose that the deletion of *sugE* in *K. pneumoniae* causes changes in the bacterial membrane structure, activating a downstream cascade to increase the production of CPS during biofilm formation. Confirmation of this hypothesis will require further investigation.

Bacteria colonize and extensively establish biofilms that cover mucosal surfaces in the gastrointestinal tract [42], [43]. In our previous study, *K. pneumoniae* PLA strain NTUH-K2044 was shown to cause liver and brain abscesses in mice infected via intragastric inoculation [23]. In the present study, we determined the competitiveness of the Δ*treC* strain in gastrointestinal tract colonization in the same murine model. The mutant displayed attenuated ability to colonize the gastrointestinal tract, suggesting that *treC* contributes to gastrointestinal tract colonization and may facilitate the pathogenesis of *K. pneumoniae*.

In conclusion, we observed higher levels of *in vitro* biofilm formation with *K. pneumoniae* PLA-associated strains than with non-tissue-invasive strains. We used the biofilm assay to identify genes that contributed to biofilm formation by the *K. pneumoniae* PLA strain. Further characterization showed that *treC* and *sugE* affected both biofilm formation and mucoviscosity, apparently by modulating CPS production. The importance of *treC* was confirmed in an *in vivo* model of gastrointestinal tract colonization, validating our screen and demonstrating that biofilm formation contributes to *K. pneumoniae* pathogenicity.

## Supporting Information

Table S1 **Primers used in this study.**
(PDF)

Table S2 **Results of** ***Klebsiella pneumoniae*** **NTUH-K2044 transposon mutants.**
(PDF)
