# Supplementary material for: Isolation of Genes Involved in Biofilm Formation of a Klebsiella pneumoniae Strain Causing Pyogenic Liver Abscess
Source: PLoS One. 2011 Aug 12;6(8):e23500. doi: 10.1371/journal.pone.0023500 (PMC3155550; doi:10.1371/journal.pone.0023500)
Supplement: Table S1 — Primers used in this study. (PDF) [file pone.0023500.s001.pdf]

Table S1. Primers used in this study.

Primers	Sequence (5’ → 3’)	Purpose	Source
Km2921R	GTACCGAGCTCGAATTCGGC	Transposon mutant sequencing	[30]
Km4180F	GTTTCATTTGATGCTCGATG	Transposon mutant sequencing	[30]
KP0416(+3)F	TACAGGCACAACGAGCAC	*sugE* deletion mutant construct	This study
KP0422(+16F)	CTATCTGGGTACCTGGTATG	*sugE* deletion mutant construct	This study
KP0420(-1)R	AAGGCTCCTTTGCTGGG	*sugE* deletion mutant construct	This study
KP0421(+534) R	CTAATCATAGCGCTGATTGAC	*sugE* deletion mutant construct	This study
KP0420(-138)F	GTCAACGGTACGGTTTAGCG	*sugE* complementation construct	This study
KP0421(+528) F	TGATTAGTGCGCGCTGATC	*sugE* complementation construct	This study
KP1237R2	AATAGCTACTCCGACGG	Complementation confirmation	[28]
KP1235F2	GCGCACGTATCGATCTC	Complementation confirmation	[28]
Kp0530-1000-F	GTGATCCCGGTGAGATG	*treC* deletion mutant construct	This study
Kp0530+1000-R	CTTCCTGTGGCTGATTGG	*treC* deletion mutant construct	This study
Kp0530-I-F	AGTACTTTCCTGCTGCAA	*treC* deletion mutant construct	This study
Kp0530-I-R	CGCTTTTCCCCGGTGGC	*treC* deletion mutant construct	This study
Kp0530-1293-F	GATTGGCTCATTCTTCCTG	*treC* deletion mutant construct	This study
Kp0530+1158-R	CAGTTAATCTCGCACTTCG	*treC* deletion mutant construct	This study
Kp0530p-F	CGATCTGCTCGATCAACT	*treC* complementation construct	This study
KP0530-R	TTATCCCTGCAGCCACCA	*treC* complementation construct	This study
Kp0530p-IF	CCTCAAAAACAGAGGGGA	*treC* complementation construct	This study
Kp0530R-IR	ACTTTCCTGCTGCAACGA	*treC* complementation construct	This study

treR-RR	AGCGGCGTAGGGAAATCG	*treB* deletion mutant construct	This study
treB-F	CCCGTTTGCGCTTTGTG	*treB* deletion mutant construct	This study
wza-1113F	CGACAGTGAATGCGTCATT	*wza* deletion mutant construct	This study
wza+1002R	TGACTGGCTTCCTGATGC	*wza* deletion mutant construct	This study
wza-IR	CCATATGTTTAAATCAGTTTTA	*wza* deletion mutant construct	This study
wza-IF 5’	AATGTCACATCATTAGTAAAC	*wza* deletion mutant construct	This study
rmpA-F	CACCAAGTATGGTTGCTCCG	*rmpA* deletion mutant construct	This study
rmpA-R	CTACGCATTCATTCTCCTTC	*rmpA* deletion mutant construct	This study
P rmpA-R	ATCCTTAATGAGCCAAATGT	*rmpA* deletion mutant construct	This study
P rmpA-F(i)	GTAAAAAAGGGGAGGGGATG	*rmpA* deletion mutant construct	This study
P rmpA-F(g)	TGGGATCAGTTCACAAGGTG	*rmpA* deletion mutant construct	This study
PrmpA-R(g)	CAAATCTTTGGAAACAAAAAG C	*rmpA* deletion mutant construct	This study
gal-145F	GCCGGGATCAAAGAGATTGTT	Expression level of *galF*	This study
gal-217R	AGGAGGTGTCGAAGTGGTTTT C	Expression level of *galF*	This study
KP3720-RF	GTCAGGTACGCCAGGATATCG	Expression level of *wza*	This study
KP3720-RR	CATCAACCTGAGGGCTTTCAA	Expression level of *wza*	This study
2707F	GAGGCCGAAGTATCCAAACT	Expression level of *wzc*	This study
2872R	CAACCTGGACATCACGAGTT	Expression level of *wzc*	This study
KP3714-RF	GCAATATGGCCAGTCCGAAA	Expression level of *magA*	This study
KP3714-RR	AAAACGTGGCTATTGCATGAT G	Expression level of *magA*	This study
wcaG-RF	TTCCACTACCCCAAACAACGA	Expression level of *wcaG*	This study
wcaG-RR	TGCGGCGCTTTCATGA	Expression level of *wcaG*	This study
manB-RF	CGCTGCGGGTCACGAT	Expression level of *manB*	This study
manB-RR	GCCGGGAGCGAAAATCA	Expression level of *manB*	This study
KP3619-202F	GGAAAATGGAGAGGGTACAA AATG	Expression level of *rmpA*	This study
KP3619-322R	CATAGATGTCATAATCACACC CTTTAGG	Expression level of *rmpA*	This study
treB-R	ATGCGCAGCTGCTCAATCT	Expression level of *treB*	This study
treB-F	CCCGTTTGCGCTTTGTG	Expression level of *treB*	This study
treC-RF	CCGACAGCGGGCAGTATT	Expression level of *treC*	This study
treC-RR	CGCCGGATTCTCCCAGTT	Expression level of *treC*	This study
licC-RF	CTGGCGCGATAAAATGTTGTA C	Expression level of *licC*	This study

licC-RR	TGCCGAAGCTGGAAATCAG	Expression level of *licC*	This study
rpoB-RF	CGTGCTGTTGCCGTTGAC	Expression level of *rpoB*	This study
rpoB-RR	GGGAAGCATCCACGTACTGAA	Expression level of *rpoB*	This study
gyrA-RF	GCGGCGATAATGTCCTTCAG	Expression level of *gyrA*	This study
gyrA-RR	GTCTCCTTCGGCATCAACATG	Expression level of *gryA*	This study
