# Supplementary material for: Isolation of Genes Involved in Biofilm Formation of a Klebsiella pneumoniae Strain Causing Pyogenic Liver Abscess
Source: PLoS One. 2011 Aug 12;6(8):e23500. doi: 10.1371/journal.pone.0023500 (PMC3155550; doi:10.1371/journal.pone.0023500)
Supplement: Table S2 — Results of Klebsiella pneumoniae NTUH-K2044 transposon mutants. (PDF) [file pone.0023500.s002.pdf]

Table S2. Results of *Klebsiella pneumoniae* NTUH-K2044 transposon mutants.

| Mutant | Probable interrupted gene (NTUH-K2044 ORF) | Function/probable function | Gene size (bp) | Tn insertion point |
|---|---|---|---|---|
| **Mutants with decreased biofilm formation** | | | | |
| Cellular processes and signaling | | | | |
| Tn4-87 | KP1_0404 | Putative secretion ATPase. Belongs to HlyD protein family involved in multidrug resistance. | 1220 | 566 |
| Tn4-97 | KP1_3573 | Polymyxin B resistance protein, *pmrD*. Prolongs the half-life of phosphorylated-*pmrA*, which is a two-component system regulator involved in modification of lipopolysaccharide and polymyxin B resistance. | 245 | 32 |
| Tn9-39 | KP1_3178 | Formate dehydrogenase H selenopolypeptide subunit, *fdhF*. Metabolism of formate under anaerobic conditions. | 2150 | 572 |
| Tn10-33 | KP1_1271 | Caseinolytic peptidase X homolog, *clpx*. A ATPase belonged to the AAA+ (ATPases associated with various cellular activities) family ATPases. Involves in the protein degradation. | 1274 | 763 |
| Tn11-19 | KP1_4552 | Putative LuxR family bacterial regulatory protein. Transcriptional regulator that involves in the regulation of quorum sensing. | 584 | 469 |
| Tn12-06 | KP1_0578 | Putative exonuclease. | 989 | 639 |
| Tn20-28 | KP1_3171 | CRISPR-associated helicase Cas3 domain-containing (HD-nuclease and DEXD helicase domain) protein. Unwinds DNA/DNA and DNA/RNA duplexes. Interferences with virus proliferation. | 2615 | 2035 |
| Surface molecules (capsule, adhesion polysaccharide, and pilin) biosynthesis | | | | |
| Tn3-2 | KP1_3720 | Putative capsular polysaccharide export protein precursor, *wza*. Capsular polysaccharide transport. | 1133 | 973 |
| Tn16-47 | KP1_0383 | Outer membrane protein, *pgaA*. Exopolysacharride biosyntheis | 2489 | 511 |
| Tn19-26 | KP1_3718 | Inner membrane tyrosin kinase, *wzc*. Capsular biosynthesis | 2153 | 1347 |
| Tn24-36 | KP1_4245 | Probable pilin chaperone, *fim*. Pilin biosynthesis. | 668 | 165 |
| Carbohydrate transport and metabolism | | | | |
| Tn10-43 | KP1_3030 | 6-phospho-beta-glucosidase A. Catalyzes the hydrolysis of 6-phospho-beta-D-glucosyl-(1,4)-D-glucose to produce D-glucose and D-glucose and 6-phosphate. Participates in glycolysis/gluconeogenesis. | 1421 | 475 |

| | | | | |
|---|---|---|---|---|
| Tn12-16 | KP1_2884 | PTS system, cellobiose-specific IIC component, *celB*. One of subunit of the membrane permease, which is responsible for the recognition and transport of the sugar across the cytoplasmic membrane. | 1280 | 615 |
| Tn16-6, Tn18-57, Tn19-70 | KP1_0530 | Trehalose-6-phosphate hydrolase, *treC*. Catalyzes the hydrolysis of trehalose-6-phosphate to produce glucose and glucose-6-phosphate. | 1655 | 1505 |
| Tn23-31 | KP1_5472 | Probable D-galactonate transport protein, *dgoT*. Belongs to Major Facilitator Superfamily. | 1337 | 180 |
| Unknown function | | | | |
| Tn4-96 | KP1_1830 | Hypothetical protein. Unknown | 122 | -1352 |
| Tn8-61 | KP1_2181 | Hypothetical protein. Unknown | 539 | 377 |
| Tn9-30 | KP1_3087 | Hypothetical protein. Unknown | 326 | 3 |
| Tn14-86 | KP1_3087 | Hypothetical protein. Unknown | 326 | 116 |
| Tn10-49 | KP_p012 | Hypothetical protein. Unknown | 995 | 180 |
| Tn21-15 | KP1_0662 | Hypothetical protein. Unknown | 2222 | 213 |
| **Mutants with increased biofilm formation** | | | | |
| Inorganic ion transport and metabolism | | | | |
| Tn10-23 | KP1_0420 | Small multidrug resistance protein, *sugE*. Confers resistance to quaternary cation compounds. | 317 | -7 |
| Transcription | | | | |
| Tn24-71 | KP1_3465 | Cold-shock protein, *cspC*. Regulates *rpoS*-dependent stress response, through stabilization of *rpoS* transcript. | 209 | -706 |
| Unknown function | | | | |
| Tn10-19 | KP1_2828 | Conserved hypothetical protein. Unknown. | 899 | 695 |
| Tn10-30 | KP1_2389 | Conserved hypothetical protein. Unknown. | 1532 | 1358 |
